# Associations of intimate partner violence with postnatal health practices in Bihar, India

**DOI:** 10.1186/s12884-017-1577-0

**Published:** 2017-11-29

**Authors:** Sabrina C. Boyce, Lotus McDougal, Jay G. Silverman, Yamini Atmavilas, Diva Dhar, Katherine Hay, Anita Raj

**Affiliations:** 1 Center on Gender Equity and Health, Department of Medicine, University of California, San Diego School of Medicine, San Diego, CA USA; 2 Bill and Melinda Gates Foundation, New Delhi, India

**Keywords:** Intimate partner violence, Post-natal health, Breastfeeding, Post-partum contraception

## Abstract

**Background:**

Reducing neonatal mortality is a global priority, and improvements in postnatal health (PNH) practices in India are needed to do so. Intimate partner violence (IPV) may be associated with PNH practices, but little research has assessed this relationship.

**Methods:**

A cross-sectional analysis of data from a representative household sample of mothers of neonates 0–11 months old in Bihar, India was conducted. The relationship between lifetime IPV experience (physical violence only, sexual violence only, or both physical and sexual violence) and PNH practices [clean cord care, kangaroo mother care, early initiation of breastfeeding (EIBF), delayed bathing, receipt of a postnatal care visit, exclusive breastfeeding, and current post-partum contraceptive use] was assessed using multivariate logistic regression.

**Results:**

Over 45% of the 10,469 mothers experienced IPV in their lifetime. The three types of IPV experiences differentially related to PNH practices. Adjusted analyses revealed that compared to those who had never experienced IPV, women who experienced *physical violence only* (29.0%) had higher odds of skin-to-skin care (AOR = 1.67, 95% CI = 1.42, 1.96) and delayed bathing (AOR = 1.19, 95% CI = 1.03, 1.37), but lower odds of EIBF (AOR = 0.81, 95% CI = 0.70, 0.93) and exclusive breastfeeding (AOR = 0.83, 95% CI = 0.71, 0.96). Mothers who had experienced *sexual violence only* (2.3%) had lower odds of practicing EIBF (AOR = 0.52, 95% CI = 0.36, 0.76). Those who had *both experiences of physical and sexual violence* (14.0%) had increased odds of postpartum modern contraceptive use (AOR = 1.35, 95% CI = 1.07, 1.71) and lower odds of delayed bathing (AOR = 0.76, 95% CI = 0.63, 0.91).

**Conclusions:**

The results of this study found differing patterns of vulnerability to poor PNH practices depending on the type of IPV experienced. Efforts to increase access to health services for women experiencing IPV and to integrate IPV intervention into such service may increase PNH practices, and as a result, reduce neonatal mortality.

## Background

The United Nation’s Sustainable Development Goals highlight neonatal mortality as a priority, setting a target of 12 deaths per 1000 births by 2030, a goal that will require substantial acceleration of progress for many countries [1]. Practices known to promote neonatal health are key to reaching this target, as evidence suggests that postnatal care within 2 days of birth, clean cord care and early initiation of breastfeeding could eliminate 30–60%, 37%, and 16%–44% of neonatal deaths, respectively [2–7]. The World Health Organization recommends that women should receive postnatal care that teaches new mothers these healthy post-natal health (PNH) practices within the first 24 hours, followed by check-ups on the second or third day, and then on the seventh day after giving birth [8, 9]. In India, one of the most populous countries in the world, and where the neonatal mortality rate is 28 per 1000 live births, national programs to strengthen maternal and neonatal health services have likely contributed to reducing the neonatal mortality rate by half over the last 15 years, yet low rates of healthy PNH practices persist, likely inhibiting further improvement [10–13]. Strengthening PNH practices in India is essential to reducing neonatal mortality.

In Bihar, one of the poorest and most populous Indian states, the neonatal mortality rate is relatively high (32.2 per 1000 live births), with neonatal mortality strongly associated with inadequate PNH practices [13]. A number of social factors are associated with PNH practices, including wealth, maternal and paternal education, caste, and religion, indicating varying PNH practices across populations [14–16]. Intimate partner violence (IPV), a human rights violation faced by more than one in three married women in India and two in five married women in Bihar, has been documented to play an important role in maternal and infant health in India and other national contexts, and may have important implications for PNH practices [11, 17–20].

Global evidence documents that women who experience IPV are less likely to engage in maternal and child health protective behaviors, including health care seeking, prior to and after pregnancy [21–23]. Studies indicate that IPV compromises women’s health practices indirectly, by inducing stress/anxiety and depression, which can impede their ability to alter their circumstances and uptake social and health services, but also directly, as abusive male partners may actively prevent women’s protective health behaviors [21, 24–26]. Less research on this topic has focused on the postnatal period specifically, despite this being a similarly critical period for maternal and child health. The limited research on IPV and the postnatal period has focused on breastfeeding practices and consistently demonstrates that IPV is associated with lower likelihood of exclusively breastfeeding infants in the first 6 months of life [27–31]. Research from India also indicates physical and sexual IPV history is associated with not using contraception postpartum [23]. Despite this evidence linking IPV and a subset of PNH practices, we found no published research from India or globally that was specific to key PNH practices beyond breastfeeding and contraceptive use, such as clean cord care, kangaroo mother care (skin-to-skin care), and delayed bathing of newborns [28, 30–32]. Nor could we identify research on the association between IPV and post-partum clinical care, although extensive research from India and elsewhere indicates that IPV is associated with delayed or no antenatal care utilization [33–35]. The current study attempts to address this significant gap in research regarding associations between IPV and post-partum health practices which may reduce neonatal mortality.

Important in the assessment of IPV as a risk factor for poor PNH practices is consideration of physical and sexual violence separately, as well as their co-occurrence, as a growing body of research suggests different forms of IPV relate to unique patterns of health practices [18, 25, 33, 34]. Previous research in India has identified that women experiencing only physical IPV are less likely than those reporting no IPV to utilize sexual health services [23, 36]. Indian women experiencing only sexual IPV have been found to be more likely to use modern contraceptives compared to women reporting no IPV [23, 34]. In the same study, however, no association was found between experiencing physical IPV alone and modern contraceptive use [23, 34].

The current study aims to elucidate the relationship between IPV, specifically when a woman has experienced physical violence only, sexual violence only, or both forms of violence, and PNH practices among a representative sample of mothers of living infants in Bihar, India. Understanding these relationships may help guide the development of new and ongoing interventions to promote neonatal survival.

## Methods

The current study includes analysis of data collected for evaluation of the Ananya program, a partnership initiated in 2012 in Bihar, India, by the Government of Bihar and the Bill and Melinda Gates Foundation designed to increase maternal and child health care utilization in the public health system using a combination of supply-side and demand generation efforts [37]. Ananya was implemented using a two-armed quasi-experimental design in which eight intervention districts were compared to the remaining 30 standard care districts. Baseline data, collected in 2012, did not include any measures of IPV, therefore the current analysis is limited to cross-sectional data from the second statewide household survey collected January to April 2014 from a representative sample of mothers of 0–11 month old infants. All data were collected by trained female study staff, subsequent to acquisition of written informed consent.

A multi-stage sampling approach was used to select villages, randomly selecting first blocks, then villages from those blocks. A listing exercise was conducted in each selected village to identify all women who had a live birth in the previous 12 months (about 13 women per village, on average). Details on study sampling and procedures are available in a previous publication by Borkum et al. [38].

The survey participation rate was 87% and yielded 11,654 completed surveys from mothers of living children 0–11 months old [38]. Women who had ever been married with a living, singleton child aged 0–11 months and were not pregnant at the time of interview were included in this analysis (*n* = 10,469); mothers of neonates requiring postnatal medical care were not excluded. Ethical approval for the original evaluation study was provided by India’s Health Ministry Screening Committee. Ethical approval for this analysis was provided by the University of California, San Diego.

### Measures

The primary independent variables were lifetime experiences of physical and sexual IPV considered as exclusive categories: no IPV, physical IPV only, sexual IPV only, sexual and physical IPV. Physical IPV was defined as experience of at least one of the following by a husband: being slapped, having an arm twisted or hair pulled, being pushed with his fist, being shaken or having something thrown at you, being kicked, dragged or beaten up, or attempted intentional choking or burning. Sexual IPV was measured by a yes/no response to, “Did your husband ever physically force you to have sexual intercourse with him even when you did not want to?” These measures have been validated in the Multi-country Study on Violence Against Women by the World Health Organization and are routinely included in Demographic and Health Surveys (DHS) [21].

Outcome variables included the following healthy newborn practices: clean cord care (nothing applied to umbilicus after cutting/tying cord); kangaroo mother care (child placed unclothed with skin to skin contact on mother’s chest/abdomen following birth); early initiation of breastfeeding (EIBF; newborn was breastfed within 1 h of birth); delayed bathing (first bath occurred 2 or more days after birth); postnatal care visit by a health worker within 48 h of birth; and exclusive breastfeeding (child received only breastmilk in 24 h prior to the survey for children <6 months; 6 months of exclusive breastfeeding reported for children 6–11 months). Current post-partum contraceptive use was defined as female or male sterilization, or current use of pill, injectable, intrauterine device (IUD), or condom. If contraceptive use was initiated post-partum but discontinued prior to the study, it was not considered current post-partum contraceptive use.

Relevant background characteristics were included as covariates: residence in an Ananya program district (yes/no); age of mother (15–19, 20–24, 25–29, 30+); age of mother at marriage (under 18 years/over 18 years old); household wealth index (quartiles; a variable constructed via principle component analysis of household characteristics and assets following the technique used in DHS); [39] mother’s education (none, primary [1–8 years], secondary [9+ years]); husband’s education (none, primary [1–8 years], secondary [9+ years]); religion/caste status (belonging to either of the minority, most-marginalized social groups in India; Muslim, scheduled caste/scheduled tribe [SC/ST], or neither); gender of focal child (male/female); parity of mother (1 birth, 2 births, 3+ births); prior neonatal death or stillbirth (one or more children stillborn or died as neonate prior to focal child/none); antenatal care visits (ANC; <4 ANC visits, 4+ ANC visits); skilled birth attendant (SBA) at birth of focal child (yes/no); age of focal child (in months); and visits of community health worker (CHW) in late pregnancy (<2/2+ CHW visits in last trimester). For analyses related to current post-partum contraceptive use, the covariate of postnatal CHW visits in which family planning was discussed (yes/no) was also included.

### Data analysis

Descriptive frequencies were calculated for all PNH practice outcomes and covariates, both overall and stratified by IPV experiences. Multivariate logistic regression models were then used to assess the association between IPV and each PNH practice, adjusting for any covariates that were significant at *p* < 0.20 levels in bivariate analyses (results not shown). All analyses were adjusted for survey design and individual sampling weights, and were conducted using Stata 13 SE (Stata Corp, College Station, TX).

## Results

More than 40% of the 10,469 mothers reported ever experiencing physical IPV by their husband (29.0% only physical violence, 14.0% physical violence accompanied by sexual violence) and more than one in six mothers reported ever experiencing sexual violence from their husband (2.3% sexual violence only, 14.0% sexual violence accompanied by physical violence) (Table 1). Almost all women (98%) who experienced IPV in their lifetimes reported recent experiences of IPV, with similar prevalence between recent and lifetime experiences (IPV in the last 12 months: physical only = 28.3%, sexual only = 2.8%, physical and sexual = 12.3%).

**Table 1:** Frequencies of maternal and neonatal care outcomes and background characteristics, overall and stratified by experiences of physical and sexual intimate partner violence (*N* = 10,469)

	Total		Lifetime experience of intimate partner violence							
			No IPV		Physical IPV only		Sexual IPV only		Physical and Sexual IPV	
	Unwtd. N	% (95% CI)	Unwtd. N	% (95% CI)	Unwtd. N	% (95% CI)	Unwtd. N	% (95% CI)	Unwtd. N	% (95% CI)
Total	10,469	–	5743	54.8% (53.0–56.5)	3019	29.0% (27.5–30.6)	241	2.3% (1.8–2.8)	1466	14.0% (12.8–15.3)
*Outcomes*										
Clean cord care										
No	7920	75.0% (73.4–76.6)	4316	74.1% (72.0–76.1)	2303	75.1% (72.3–77.7)	186	77.8% (68.7–84.8)	1115	78.0% (74.5–81.1)
Yes	2549	25.0% (23.4–26.6)	1427	25.9% (23.9–28.0)	716	24.9% (22.3–27.7)	55	22.2% (15.2–31.3)	351	22.0% (18.9–25.5)
Skin-to-skin care										
No	6935	65.4% (63.0–67.7)	3944	67.8% (64.9–70.5)	1781	57.9% (54.3–61.3)	154	63.0% (52.9–72.1)	1056	71.8% (67.1–76.2)
Yes	3534	34.6% (32.3–37.0)	1799	32.2% (29.5–35.1)	1238	42.1% (38.7–45.7)	87	37.0% (27.9–47.1)	410	28.2% (23.8–32.9)
Early initiation of breastfeeding										
No	5443	51.8% (50.0–53.7)	2900	49.3% (47.1–51.5)	1629	54.5% (51.5–57.5)	139	65.4% (56.8–73.1)	775	54.0% (49.4–58.6)
Yes	5026	48.2% (46.3–50.0)	2843	50.7% (48.5–52.9)	1390	45.5% (42.5–48.5)	102	34.6% (26.9–43.2)	691	46.0% (41.4–50.6)
Delay of first bath										
No	4394	42.6% (40.7–44.6)	2414	41.7% (39.4–44.1)	1198	40.2% (37.4–43.1)	105	47.7% (38.5–57.1)	677	50.5% (46.1–54.9)
Yes	6075	57.4% (55.4–59.3)	3329	58.3% (55.9–60.6)	1821	59.8% (56.9–62.6)	136	52.3% (42.9–61.5)		49.5% (45.1–53.9)
Postnatal care within 48 h										
No	9105	85.9% (84.5–87.1)	5038	86.8% (85.3–88.2)	2607	84.8% (82.3–86.9)	200	82.7% (71.8–90.0)	1260	84.9% (81.2–88.1)
Yes	1364	14.1% (12.9–15.5)	705	13.2% (11.8–14.7)	412	15.2% (13.1–17.7)	41	17.3% (10.0–28.2)	206	15.1% (11.9–18.8)
Exclusive breastfeeding										
No	3400	33.0% (31.6–34.4)	1832	31.3% (29.3–33.3)	1011	35.5% (33.1–37.9)	87	37.6% (28.4–47.7)	470	33.8% (30.2–37.6)
Yes	7069	67.0% (65.6–68.4)	3911	68.7% (66.7–70.7)	2008	64.5% (62.1–66.9)	154	62.4% (52.3–71.6)	996	66.2% (62.4–69.8)
Post-partum contraception										
No	8835	85.3% (84.0–86.5)	4894	85.9% (84.2–87.4)	2562	86.8% (85.0–88.4)	202	86.7% (80.7–91.1)	1177	79.4% (75.8–82.7)
Yes	1634	14.7% (13.5–16.0)	849	14.1% (12.6–15.8)	457	13.2% (11.6–15.0)	39	13.3% ([8.9–19.3)	289	20.6% (17.3–24.2)
*Background characteristics*										
Ananya district										
No	7833	76.9% (74.0–79.7)	4250	75.6% (72.2–78.7)	2281	78.0% (74.3–81.2)	194	84.5% (76.8–90.0)	1108	78.8% (74.3–82.7)
Yes	2636	23.1% (20.3–26.0)	1493	24.4% (21.3–27.8)	738	22.0% (18.8–25.7)	47	15.5% (10.0–23.2)	358	21.2% (17.3–25.7)
Age										
15–19	391	4.1% (3.5–4.9)	233	4.5% (3.6–5.6)	98	3.8% (2.8–5.3)	11	2.9% (1.1–7.7)	49	3.5% (2.5–5.0)
20–24	4413	42.3% (40.7–44.0)	2552	44.2% (42.1–46.3)	1176	39.3% (36.5–42.2)	99	45.0% (35.4–54.9)	586	40.9% (37.1–44.8)
25–29	3905	35.9% (34.2–37.5)	2120	36.3% (34.1–38.5)	1137	35.3% (32.7–38.0)	99	41.5% (32.6–51.0)	549	34.4% (30.8–38.1)
30+	1760	17.7% (16.4–19.0)	838	15.0% (13.5–16.7)	608	21.5% (19.3–23.9)	32	10.6% (6.5–17.0)	282	21.2% (18.0–24.7)
Age at marriage										
< 18	4594	45.1% (43.1–47.1)	2162	39.4% (37.2–41.7)	1587	52.6% (49.5–55.7)	118	52.1% (41.7–62.2)	727	50.5% (46.2–54.8)
18+	5875	54.9% (52.9–56.9)	3581	60.6% (58.3–62.8)	1432	47.4% (44.3–50.5)	123	47.9% (37.8–58.3)	739	49.5% (45.2–53.8)
Wealth quartile										
1 (poorest)	2877	28.6% (26.5–30.8)	1402	25.8% (23.5–28.1)	989	34.4% (30.9–37.9)	62	21.8% (15.6–29.7)	424	29.0% (24.8–33.5)
2	2140	21.3% (19.9–22.8)	1129	21.7% (20.0–23.6)	670	21.8% (19.5–24.3)	38	18.0% (12.0–26.1)	303	19.2% (16.3–22.6)
3	2509	24.7% (23.3–26.2)	1293	23.3% (21.5–25.2)	733	24.2% (21.8–26.7)	72	38.0% (28.1–49.0)	411	29.2% (25.5–33.2)
4 (wealthiest)	2943	25.4% (23.5–27.3)	1919	29.3% (26.9–31.8)	627	19.7% (17.2–22.4)	69	22.1% (14.7–31.9)	328	22.6% (18.8–26.9)
Education										
None	5351	53.1% (50.9–55.2)	2569	47.6% (45.1–50.1)	1817	62.0% (59.1–64.9)	112	45.8% (36.4–55.5)	853	56.9% (52.4–61.3)
Primary	2705	25.9% (24.4–27.4)	1520	26.8% (25.0–28.7)	740	23.3% (21.0–25.8)	68	30.2% (23.4–38.0)	377	26.7% (22.8–31.0)
Secondary	2413	21.1% (19.6–22.7)	1654	25.5% (23.5–27.7)	462	14.7% (12.7–16.9)	61	24.0% (17.7–31.8)	236	16.4% (13.3–20.0)
Spouse’s education										
None	3237	32.4% (30.5–34.4)	1518	28.5% (26.4–30.8)	1132	39.5% (36.6–42.5)	69	23.3% (16.8–31.5)	518	34.4% (30.8–38.2)
Primary	3504	33.9% (32.5–35.3)	1877	33.0% (31.2–34.9)	1045	35.6% (33.3–38.1)	74	33.3% (24.5–43.4)	508	33.9% (30.6–37.4)
Secondary	3728	33.7% (31.7–35.7)	2348	38.5% (36.0–41.0)	842	24.9% (22.1–27.8)	98	43.4% (33.3–54.0)	440	31.7% (27.7–35.9)
Caste/religion										
Neither SC/ST nor Muslim	5963	56.8% (53.7–59.8)	3507	61.1% (57.5–64.6)	1558	51.4% (47.8–55.1)	149	63.3% (52.8–72.7)	749	49.8% (44.3–55.3)
SC/ST	2693	26.0% (23.5–28.6)	1178	21.0% (18.5–23.8)	991	33.2% (29.7–36.9)	53	19.0% (12.6–27.7)	471	31.4% (26.7–36.5)
Muslim	1813	17.2% (14.6–20.3)	1058	17.8% (14.8–21.3)	470	15.4% (12.5–18.8)	39	17.7% (11.4–26.5)	246	18.8% (14.5–24.1)
Gender of focal child										
Female	4857	46.4% (45.0–47.9)	2663	46.3% (44.5–48.2)	1439	47.6% (44.8–50.4)	109	39.8% (31.4–48.8)	646	45.4% (41.6–49.3)
Male	5612	53.6% (52.1–55.0)	3080	53.7% (51.8–55.5)	1580	52.4% (49.6–55.2)	132	60.2% (51.2–68.6)	820	54.6% (50.7–58.4)
Parity										
1	3239	30.9% (29.5–32.4)	2036	35.0% (33.2–36.8)	778	27.0% (24.6,29.5)	69	29.5% (20.5–40.5)	356	23.4% (20.5–26.7)
2	2979	27.7% (26.4–29.1)	1663	28.2% (26.6–29.8)	827	26.5% (24.1–29.2)	72	32.2% (23.8–42.0)	417	27.8% (24.2–31.6)
3+	4251	41.3% (39.8–42.9)	2044	36.8% (35.0–38.7)	1414	46.5% (43.7–49.3)	100	38.3% (29.9–47.5)	693	48.8% (45.1–52.5)
Age of child (months) ^a^	–	5.0 (4.9–5.1)	–	4.9 (4.8–5.1)	–	5.1 (5.0–5.3)	–	4.9 (4.3–5.6)	–	5.2 (5.0–5.4)
Previous neonatal death or stillbirth										
No	9588	91.3% (90.4–92.2)	5336	93.0% (91.9–93.9)	2741	89.8% (88.1–91.4)	216	84.6% (76.1–90.5)	1295	89.2% (86.4–91.5)
Yes	881	8.7% (7.8–9.6)	407	7.0% (6.1–8.1)	278	10.2% (8.6–11.9)	25	15.4% (9.5–23.9)	171	10.8% (8.5–13.6)
4 or more antenatal care visits										
No	8400	81.2% (79.7–82.5)	4459	78.2% (76.2–80.1)	2524	85.5% (83.7–87.2)	192	80.4% (73.0–86.2)	1225	83.7% (81.0–86.2)
Yes	2069	18.8% (17.5–20.3)	1284	21.8% (19.9–23.8)	495	14.5% (12.8–16.3)	49	19.6% (13.8–27.0)	241	16.3% (13.8–19.0)
Skilled birth attendance										
No	2826	28.0% (26.1–30.0)	1382	24.9% (22.8–27.1)	958	32.6% (29.5–36.0)	65	29.3% (21.9–38.1)	421	30.4% (26.6–34.5)
Yes	7643	72.0% (70.0–73.9)	4361	75.1% (72.9–77.2)	2061	67.4% (64.0–70.5)	176	70.7% (61.9–78.1)	1045	69.6% (65.5–73.4)
2+ CHW visits in final trimester										
No	7148	67.1% (65.0–69.1)	4060	69.3% (66.6–71.9)	1969	64.4% (61.0–67.7)	159	59.9% (50.3–68.8)	960	65.0% (60.2–69.5)
Yes	3321	32.9% (30.9–35.0)	1683	30.7% (28.1–33.4)	1050	35.6% (32.3–39.0)	82	40.1% (31.2–49.7)	506	35.0% (30.5–39.8)
Postnatal CHW visit discussing family planning										
No	9184	87.3% (85.9–88.6)	5214	90.2% (88.6–91.7)	2556	84.3% (81.6–86.6)	206	85.5% (78.7–90.4)	1208	82.4% (78.0–86.0)
Yes	1285	12.7% (11.4–14.1)	529	9.8% (8.3–11.4)	463	15.7% (13.4–18.4)	35	14.5% (9.6–21.3)	258	17.6% (14.0–22.0)

^a^Mean (95% confidence interval)

The majority of participants (78.2%) were between the ages of 20–29 and more than two-thirds had at least two children (Table 1). Nearly half of women (45.1%) were under age 18 when they married, 53.1% received no education, and 43.2% belonged to a marginalized religion/caste. Healthy newborn practices of delayed bathing (57.4%), early initiation of breastfeeding (48.2%) and exclusive breastfeeding (67.0%) were practiced by a moderate proportion of the sample, while clean cord care (25.0%), skin-to-skin care (34.6%), receiving postnatal care within 48 h (14.1%), and current post-partum contraceptive use (14.7%) were less common.

Experiencing physical IPV only was disproportionately prevalent among mothers who practiced skin-to-skin care (42.1% vs. 32.2% no IPV), were age 30+ (21.5% vs. 15% no IPV), were under age 18 when they married (52.6% vs. 39.4% of those experiencing no IPV), in the lowest wealth quartile (34.4% vs. 25.8% no IPV), had no education (62.0% vs. 47.6% no IPV), had a spouse with no education (39.5% vs. 28.5% no IPV), were from SC/ST caste (33.2% vs. 21.0% no IPV), had three or more children (46.5% vs. 36.8% no IPV), received fewer than 4 ANC visits (85.5% vs. 78.2% no IPV), did not have a skilled birth attendant (32.6% vs. 24.9% no IPV) and received a postnatal visit to discuss family planning (15.7% vs. 9.8% no IPV) (Table 1). Sexual violence only was reported to be highly prevalent among mothers who did not practice EIBF (65.4% vs. 49.3% of those experiencing no IPV) and were in the third highest wealth quartile (38.0% vs. 23.3% no IPV). Women reporting both physical and sexual violence were disproportionately represented among those reporting no delayed bathing (50.5% vs. 41.7% no IPV), post-partum contraceptive use (20.6% vs. 14.1% no IPV), age 30+ (21.2% vs. 15% no IPV), under age 18 when they got married (50.5% vs. 39.4% no IPV), being in the lowest and second highest wealth quartiles (lowest quartile: 29.0% vs. 25.8% no IPV; third quartile: 29.2% vs. 23.3% no IPV), no education (56.9% vs. 47.6% no IPV), being from SC/ST caste (31.4% vs. 21.0% no IPV), three or more previous births (48.8% vs. 36.8% no IPV), less than 4 ANC (83.7% vs. 78.2% no IPV), and having had a postnatal visit to discuss family planning (17.6% vs. 9.8% no IPV).

### Multivariate models to assess association between IPV and PNH practices

Adjusted analyses revealed that, compared to those who had never experienced physical or sexual IPV, women who experienced physical IPV only had higher odds of skin-to-skin care (AOR = 1.67, 95% CI = 1.42, 1.96) and delayed bathing (AOR = 1.19, 95% CI = 1.03, 1.37), but lower odds of EIBF (AOR = 0.81, 95% CI = 0.71, 0.93), and exclusive breastfeeding (AOR = 0.83, 95% CI = 0.71, 0.96) (see Table 2). Those who experienced sexual IPV only, relative to those who had experienced neither form of IPV, had lower odds of practicing EIBF (AOR = 0.52, 95% CI = 0.36, 0.76). Women who experienced both physical and sexual IPV had increased odds of currently using a modern contraceptive method (AOR = 1.35, 95% CI = 1.07, 1.71) and lower odds of delayed bathing (AOR = 0.76, 95% CI = 0.63, 0.91).

**Table 2:** Multivariate logistic regression models showing relationship between intimate partner violence and postnatal care behaviors and services (n = 10,469)

	Clean cord care	Skin-to-skin care	Early initiation of breastfeeding	Delay of first bath	Postnatal care within 48 h	Exclusive breastfeeding	Post-partum contraception
Lifetime experience of intimate partner violence							
Never	REF	REF	REF	REF	REF	REF	REF
Physical only	0.92 (0.78–1.08)	1.66 (1.41–1.96)***	0.81 (0.71–0.93)**	1.19 (1.03–1.37)*	1.16 (0.95–1.42)	0.83 (0.71–0.96)*	0.82 (0.68–0.998)*
Sexual only	0.91 (0.58–1.41)	1.33 (0.85–2.06)	0.52 (0.36–0.76)***	0.87 (0.60–1.26)	1.22 (0.65–2.29)	0.74 (0.49–1.12)	0.91 (0.56–1.46)
Physical and sexual	0.82 (0.66–1.02)	0.87 (0.68–1.12)	0.83 (0.67–1.01)	0.76 (0.63–0.91)**	1.14 (0.85–1.55)	0.92 (0.75–1.15)	1.35 (1.07–1.71)*
Ananya district							
No	REF	REF	REF	REF	–	REF	REF
Yes	1.52 (1.24–1.85)***	1.53 (1.25–1.88)***	1.21 (1.04–1.41)*	1.53 (1.29–1.82)***	–	1.16 (1.00–1.34)*	1.48 (1.18–1.86)**
Age							
15–19	–	REF	REF	REF	–	–	REF
20–24	–	0.90 (0.60–1.36)	1.44 (1.10–1.89)**	0.72 (0.50–1.04)	–	–	0.92 (0.58–1.44)
25–29	–	0.77 (0.50–1.19)	1.49 (1.11–2.00)**	0.66 (0.44–0.99)*	–	–	0.91 (0.56–1.48)
30+	–	0.97 (0.62–1.52)	1.49 (1.10–2.02)**	0.75 (0.50–1.11)	–	–	1.02 (0.65–1.61)
Age at marriage							
< 18	REF	REF	REF	REF	–	REF	REF
18+	1.40 (1.20–1.63)***	1.15 (0.98–1.34)	1.17 (1.04–1.32)**	1.18 (1.04–1.34)**	–	1.14 (0.99–1.32)	1.06 (0.89–1.25)
Wealth quartile							
1 (poorest)	REF	REF	–	REF	REF	REF	REF
2	0.96 (0.80–1.16)	1.08 (0.88–1.34)	–	0.85 (0.72–1.01)	1.13 (0.85–1.50)	1.06 (0.87–1.29)	1.12 (0.89–1.41)
3	0.80 (0.68–0.96)*	1.27 (1.00–1.60)*	–	0.88 (0.74–1.04)	1.18 (0.91–1.54)	0.92 (0.76–1.11)	1.38 (1.11–1.72)**
4 (wealthiest)	0.92 (0.71–1.18)	1.30 (1.04–1.62)*	–	0.89 (0.73–1.08)	0.90 (0.67–1.22)	0.84 (0.69–1.03)	1.43 (1.13–1.82)**
Education							
None	REF	–	REF	REF	–	–	–
Primary	0.95 (0.80–1.12)	–	1.16 (1.02–1.34)*	1.04 (0.89–1.20)	–	–	–
Secondary	0.92 (0.73–1.15)	–	0.85 (0.73–1.00)	0.98 (0.81–1.20)	–	–	–
Spouse’s education							
None	REF	–	–	REF	–	REF	–
Primary	0.85 (0.71–1.03)	–	–	0.94 (0.80–1.09)	–	0.84 (0.72–0.98)*	–
Secondary	0.72 (0.59–0.89)**	–	–	1.01 (0.85–1.20)	–	0.92 (0.77–1.10)	–
Caste/religion							
Neither SC/ST nor Muslim	–	REF	REF	REF	–	–	REF
SC/ST	–	1.04 (0.85–1.27)	0.94 (0.81–1.11)	0.85 (0.71–1.02)	–	–	1.00 (0.80–1.24)
Muslim	–	0.79 (0.62–1.00)	0.77 (0.63–0.95)*	1.09 (0.91–1.30)	–	–	0.59 (0.45–0.78)***
Gender of focal child							
Female	–	–	–	–	–	–	REF
Male	–	–	–	–	–	–	1.17 (0.99–1.39)
Parity							
1	–	REF	REF	REF	–	REF	REF
2	–	0.97 (0.83–1.13)	1.29 (1.10–1.51)**	1.05 (0.90–1.23)	–	1.25 (1.06–1.47)**	1.75 (1.37–2.25)***
3+	–	0.91 (0.77–1.09)	1.26 (1.05–1.52)*	1.08 (0.90–1.29)	–	1.18 (1.01–1.37)*	3.39 (2.61–4.40)***
Age of child (months)^1^	0.97 (0.95–0.99)*	–	–	0.98 (0.96–0.999)	–	0.78 (0.77–0.80)	1.08 (1.06–1.11)***
Previous neonatal death or stillbirth							
No	–	REF	REF	REF	–	–	–
Yes	–	0.88 (0.68–1.13)	0.66 (0.54–0.82)	0.88 (0.71–1.08)	–	–	–
4 or more antenatal care visits							
No	REF	–	–	REF	REF	REF	REF
Yes	0.82 (0.67–1.01)	–	–	1.16 (0.999–1.35)	1.32 (1.05–1.66)*	0.81 (0.68–0.97)*	1.23 (1.01–1.49)*
Skilled birth attendance							
No	REF	REF	REF	REF	REF	REF	REF
Yes	0.81 (0.70–0.94)**	1.54 (1.29–1.84)***	1.19 (1.03–1.37)*	1.92 (1.66–2.21)***	1.82 (1.37–2.41)***	1.21 (1.04–1.39)*	1.38 (1.12–1.70)**
2+ CHW visits in final trimester							
No	–	REF	REF	–	REF	–	REF
Yes	–	1.27 (1.07–1.51)**	1.26 (1.11–1.44)***	–	4.22 (3.46–5.15)***	–	0.88 (0.72–1.06)
Postnatal CHW visit discussing family planning							
No	N/A	N/A	N/A	N/A	N/A	N/A	REF
Yes	N/A	N/A	N/A	N/A	N/A	N/A	2.34 (1.88–2.92)***

Models show adjusted odds ratios (95% confidence intervals), and include intimate partner violence as well as any covariates significant at the p < 0.20 level in bivariate models

*p < 0.05

**p < 0.01

***p < 0.001

^1^Mean (95% confidence interval)

Based on previous work and observed effects in this study, current post-partum contraceptive use was further explored in post-hoc analyses [34]. A multinomial regression assessed the association between IPV and specific types of contraception (sterilization [male or female], pills, condoms, or “other”, which included IUD and injectable contraception), and included all covariates used in the main multivariate model for current modern contraception.

Post-hoc exploratory analysis identified that of the 1274 women using post-partum modern contraceptives, 119 (9.3%) were using male or female sterilization, 31 (2.4%) oral contraception (pills), 62 (4.9%) condoms, and 11 (0.9%) other methods for preventing pregnancy. A multinomial regression assessing the association between IPV and method-specific current contraceptive use found that, relative to women who had not experienced IPV, women experiencing physical IPV only were less likely to be using oral contraception (adjusted relative risk ratio [ARRR] = 0.53, 95% CI = 0.32, 0.87, *p* = 0.01) and women experiencing both physical and sexual IPV were twice as likely to be using condoms (ARRR = 2.04, 95% CI = 1.43, 2.92, *p* < 0.001) (Table 3). Different patterns of association of type of IPV with sterilization and with other forms of contraception were not observed.

**Table 3:** Post-hoc multinomial regression model assessing relationship between intimate partner violence and type of current post-partum contraception use

	Current postpartum contraception			
	Sterilization (male or female)	Pill	Condom	Other^
Lifetime experience of IPV				
No IPV ever	REF	REF	REF	REF
Physical IPV only	0.89 (0.70–1.13)	0.53 (0.32–0.87)*	0.95 (0.67–1.33)	0.60 (0.27–1.36)
Sexual IPV only	0.97 (0.49–1.92)	1.00 (0.37–2.67)	0.94 (0.47–1.89)	0.25 (0.05–1.23)
Physical and sexual IPV	1.13 (0.82–1.55)	1.17 (0.64–2.13)	2.04 (1.43–2.92)***	1.23 (0.52–2.90)

Multinomial regression model shows adjusted relative risk ratios (95% confidence intervals), and adjusts for all covariates shown in the post-partum contraception model in Table 2. Reference category is no current contraception

^Includes IUD and injectables

*p < 0.05

***p < 0.001

## Discussion

Findings from this study indicate that almost half of women in Bihar, India have experienced physical and/or sexual violence from their husband. One in six women reported sexual violence, most often accompanied by physical violence. Associations between IPV and PNH practices were found, including healthy breastfeeding practices, skin to skin care, delayed bathing, and postpartum contraception use, but suggest a complex risk pattern across types of IPV.

The National Family Health Survey-4 (NFHS-4) conducted in 2015/16 found a similar rate of spousal violence among ever-married women in Bihar (43.2%) [17]. Sexual violence was (16.3%) was also common, with only 2% of women experiencing sexual violence without physical violence. This prevalence reflects that reported for Bihar in the NFHS-3 conducted in 2005/6 (NFHS-4 data not yet available) [11].

Intimate partner violence was negatively associated with healthy breastfeeding practices. The odds of early initiation of breastfeeding were decreased by between 19% and 48% among women who experienced physical or sexual IPV, respectively. This pattern is consistent with earlier studies of the association of types of IPV and breastfeeding [28, 32, 40, 41].

Odds of exclusive breastfeeding for 6 months were 17% lower among women reporting physical IPV only. Uniform with earlier studies, these results could possibly indicate limited autonomy of mothers to make breastfeeding decisions, lower confidence to be able to breastfeed, or resistance to the intimate personal contact involved in breastfeeding related to trauma [27, 28, 31, 32, 40–44].

A novel finding from this study is that women who reported only physical violence were more likely to enact certain PNH practices, including skin-to-skin care and delayed bathing. A possible explanation for this relationship may be that, while experiencing physical IPV may force a woman to compromise her own health and self-care, she may work even harder than her peers to provide adequate care for her infant as a way of compensating for any disruptions her husband’s IPV may be causing within the family [45]. In contrast with these findings, women experiencing a combination of physical and sexual violence appear to be less able to enact these same PNH behaviors. Previous research in Brazil has documented higher risk of postpartum depression associated with the co-occurrence of multiple forms of violence during pregnancy [46]. This previous work may shed some light on why the present study observed lower rates of PNH behaviors among women experiencing multiple forms of violence. The opposing effects seen in this sample for these PNH practices for different types of IPV may have important implications for understanding how the context of IPV contributes to health practices and warrant further study. Further work in this area should consider assessment of motivations for and barriers to these practices in the context of IPV, particularly in the case of delayed bathing which is affected by both culture and access to soap and water, where skin-to-skin contact requires primarily bodily resources, such as time and energy, from the mother.

Likelihood of postpartum contraception also differed across IPV experiences. Women who reported experiencing only physical IPV had 18% lower odds of postpartum contraception, while women reporting both sexual and physical IPV had a 35% increased odds of postpartum contraception. Significantly lower postpartum oral contraception use among women experiencing physical IPV only and significantly higher postpartum condom use among women experiencing physical and sexual IPV was observed. These findings were unexpected in light of prior research conducted with representative samples in India. These method-specific results differed from prior findings from national data of married women in India that found lower condom but higher oral contraception usage among women experiencing sexual violence [34]. Raj et al. found that in a situation where women have little control over sex (i.e. experience forced sex), they are actually more likely to use contraception compared to women not experiencing forced sex, suggesting that they may be using contraception to gain greater control of their reproductive health in the face of loss of control over sex [23, 47]. This previous study was not limited to contraception use during the postpartum period [34]. Further research is needed to confirm and explore the results found here, and to understand if this is a changing dynamic around contraception use in India or if this is specific to postpartum use.

Sociodemographic characteristics were also found to relate to beneficial PNH behaviors. Being age 18 or older when married and at the time of survey, and having had a child prior to the index child were all positively associated with PNH behaviors, particularly those related to breastfeeding. Higher wealth was associated with increased odds of applying nothing to the umbilicus after cutting/tying the cord, skin-to-skin care, and current post-partum contraceptive. An unexpected trend, however, was that as spousal education increased, adherence to clean cord care was less likely. More research is needed to understand this association, as spousal education may be a marker for a particular set of husband-related characteristics that are associated with a lack of support for this PNH practice or behaviors related to neonatal care that counteract these beneficial PNH practices.

Exposure to health services, including ANC, SBA, and CHW visits during the last trimester of pregnancy, emerged as experiences largely beneficial to enactment of PNH practices, even in models adjusted for IPV and other intervention services. However, SBA and four or more ANC were less likely among women experiencing physical IPV. Efforts to ensure that women who are experiencing IPV are able to access health services are needed. Women living in an Ananya program district, a program that aims to increase access to and quality of maternal and child health services, had higher odds of all assessed PNH practices, except for postnatal care, regardless of IPV exposure. Postnatal care within 48 h of childbirth, though, was not reduced among women exposed to IPV. This finding suggests that beyond being of benefit for neonates of all women, postnatal health services may be an important “touch-point” for victims of IPV, particularly in the past year. Programs like Ananya, and health services such as postnatal care, may offer a potentially important opportunity to provide support and intervene with households to help reduce or mitigate their exposure to IPV and related health vulnerabilities for both new mothers and their neonates [38]. The Ananya program includes the use of community health workers to conduct household visits during pregnancy to provide basic services and facilitate access to clinical care, an intervention model upon which it might be possible to build in services to address IPV in relation to postnatal care practices.

Consideration of the limitations of this study is important for interpreting results. Data analyzed in this study are the second cross-section of women who have recently given birth from an evaluation of a large-scale effort to improve health services, and results demonstrate a positive effect on PNH practices for those residing in the intervention districts. To address potential intervention effects on the currently assessed associations, all multivariate analyses were adjusted for exposure to this intervention. Additionally, unobserved confounding factors not accounted for may obscure true effects, though models were adjusted for a variety of highly relevant covariates in order to limit this potential. While reports of past 12 month IPV experiences likely temporarily predicate or overlap with the postnatal period in which PNH practices may have occurred, these results only provide indication of correlation and do not indicate causal relationships. One important limitation of these data is that they rely on participant self-report of PNH practices and IPV experiences from up to 11 months post-partum. This relatively long time gap may leave data vulnerable to recall and desirability bias, which could influence results to be biased either toward or away from the null hypothesis. Additionally, this analysis of a dichotomous measure of IPV experience does not provide any information on how frequency of IPV could change the association between IPV and PNH practices. The results of this study are representative of recent mothers in the state of Bihar, and cannot be generalized to other populations or regions of India or elsewhere.

## Conclusion

The current study found a high prevalence of IPV, including recent IPV, among this representative sample of recent mothers. Results indicate different patterns of vulnerability to poor PNH practices depending on the type of IPV experienced, whether physical only, sexual only, or both forms. Overall, IPV was found to be largely associated with poor PNH practices, most clearly demonstrated in its effects on breast feeding. However, a smaller subset of beneficial PNH practices were more likely among women based on the type of IPV experienced. Physical violence alone appeared to be associated with mothers’, perhaps, greater efforts to ensure care for their neonate, whereas experiences of both physical and sexual IPV, or of only sexual IPV, appeared to significantly inhibit healthy PNH practices. More research is needed to further clarify the observed associations and mechanisms behind these.

The pattern of IPV experience and postpartum contraception use is unclear. The overall association between IPV and contraception use mirrored findings from other studies, but the type-specific analyses of postpartum contraception use suggests a potentially changing dynamic for the association between contraception type and type of IPV experience or a unique dynamic for postpartum contraception use.

Opportunity for mitigating the negative impact of IPV on PNH behaviors may exist within health care encounters, particularly within postnatal care. Current governmental efforts to increase access and quality of maternal and neonatal health services may also facilitate women experiencing IPV to engage with PNH practices, and as a result, reduce neonatal mortality. Moreover, postnatal health care visits may provide an important opportunity for providing IPV support to victims, which could be built into existing governmental efforts to strengthen quality of care, to reduce IPV and its impact on maternal and neonatal health.

## Abbreviations

ANC: Antenatal care
AOR: Adjusted odds ratio
ARRR: Adjusted relative risk ratio
CHW: Community health worker
DHS: Demographic and Health Surveys
EIBF: Early initiation of breastfeeding
IPV: Intimate partner violence
IUD: Intrauterine device
PNH: Postnatal health
SBA: Skilled birth attendant
SC/ST: Scheduled caste/scheduled tribe
